# Dynamic Adjustment of Stimuli in Real Time Functional Magnetic Resonance Imaging

**DOI:** 10.1371/journal.pone.0117942

**Published:** 2015-03-18

**Authors:** I. Jung Feng, Anthony I. Jack, Curtis Tatsuoka

**Affiliations:** 1 Case Western Reserve University, Department of Epidemiology and Biostatistics, Cleveland, Ohio, United States of America; 2 Case Western Reserve University, Department of Cognitive Science, Cleveland, Ohio, United States of America; 3 Case Western Reserve University, Department of Neurology, Cleveland, Ohio, United States of America; Max Planck Institute for Human Cognitive and Brain Sciences, GERMANY

## Abstract

The conventional fMRI image analysis approach to associating stimuli to brain activation is performed by carrying out a massive number of parallel univariate regression analyses. fMRI blood-oxygen-level dependent (BOLD) signal, the basis of these analyses, is known for its low signal-noise-ratio and high spatial and temporal signal correlation. In order to ensure accurate localization of brain activity, stimulus administration in an fMRI session is often lengthy and repetitive. Real-time fMRI BOLD signal analysis is carried out as the signal is observed. This method allows for dynamic, real-time adjustment of stimuli through sequential experimental designs. We have developed a voxel-wise sequential probability ratio test (SPRT) approach for dynamically determining localization, as well as decision rules for stopping stimulus administration. SPRT methods and general linear model (GLM) approaches are combined to identify brain regions that are activated by specific elements of stimuli. Stimulus administration is dynamically stopped when sufficient statistical evidence is collected to determine activation status across regions of interest, following predetermined statistical error thresholds. Simulation experiments and an example based on real fMRI data show that scan volumes can be substantially reduced when compared with pre-determined, fixed designs while achieving similar or better accuracy in detecting activated voxels. Moreover, the proposed approach is also able to accurately detect differentially activated areas, and other comparisons between task-related GLM parameters that can be formulated in a hypothesis-testing framework. Finally, we give a demonstration of SPRT being employed in conjunction with a halving algorithm to dynamically adjust stimuli.

## Introduction

Functional neuroimaging technology is widely used for clinical applications, such as determining early-stage disease by detecting abnormal functioning in specific brain regions [1,2], assessing treatment effectiveness [3], and for pre-surgical assessments to help prevent resectioning that can damage important cognitive and motor functions [3]. Functional magnetic resonance imaging (fMRI) provides neural images with high spatial resolution by non-invasively detecting task-related blood-oxygen-level-dependent (BOLD) signal changes which are associated with neural activity in the brain [4]. Importantly, then, fMRI allows for precise localization of brain functioning.

However, fMRI signals present analytical challenges because they are abundant, noisy, and highly correlated, both spatially and temporally. One whole brain fMRI image at a given time involves simultaneous BOLD measurement of hundreds of thousands voxels, with recordings taken at each voxel every repetition time (TR) unit. Even when a voxel is indeed activated in relation to a task, the associated task-related signal may only involve 0.5 to 2% signal change in measured BOLD signal [3]. In order to ensure accurate spatial localization to overcome low signal-to-noise-ratio (SNR), and to overcome the possibility of large, unexpected disruptions to experimentation such as sudden eye or head movements, redundant and lengthy fMRI sessions are usually implemented. Moreover, variability that may affect SNR may differ depending on the individual. Conservative assumptions about potential variability may need to be made in order to help insure that an experimental design will provide sufficient information about localization of activation for a large proportion of subjects. This leads to inefficient designs and high costs for fMRI, and exposes the signal data to fatigue and learning effects.

With progress in fMRI acquisition and computational processing, it has become feasible to observe brain activity during experimentation. This advance is known as real-time fMRI (rtfMRI) [5,6,7,8]. This advent of real-time processing of BOLD signals creates an important opportunity: the ability to adapt experimental stimuli in real-time according to individual response and variability. This will enhance fMRI experimentation, by making it more efficient through reducing redundancies. For instance, if through real-time signal processing the activation status of voxels within a region of interest (ROI) becomes clear, experimentation can be terminated early without having to fully proceed through a pre-determined sequence of stimuli. As a result, precise classification of neural activity can be obtained while requiring less fMRI scan time as compared to conventional fMRI experimentation.

To date, we are not aware of any other studies that have focused on applying sequential experimental design approaches on fMRI. Dynamic adjustment of stimuli via sequential analysis, applied on analyzing real time fMRI signal, is thus considered here. Sequential analysis involves making statistically based evaluations after collection of each new observation. As a fundamental first step in developing a new generation of dynamic fMRI stimuli, focus here will be given on approaches to dynamic stopping rules for experimental administration. The statistical setting is the standard general linear model (GLM) framework commonly used in fMRI, where regression parameters are associated to specific tasks, and statistical inference of activation status depends on whether or not the parameter has value zero. Sampling is stopped when accumulated data is able to provide sufficient information to make a decision under pre-defined Type I and Type II errors. For hypothesis testing under general conditions, it has been demonstrated that Wald’s sequential probability ratio test (Wald’s SPRT) requires minimum expected sample size compared with all other fixed sample and sequential tests [9]. Here, Wald’s SPRT is applied as the foundation of future work in dynamic adjustment of stimuli for seeking more efficient experimental fMRI designs using rtfMRI.

The proposed approach allows for dynamically deciding whether a voxel is active or not, to a pre-specified activation level. In aggregate, voxel-wise analysis is simultaneously conducted across an ROI, to dynamically detect activation patterns. This requires “global level” stop rules that takes into account how decisively classification is being conducted across voxels, which will be proposed. It will be seen that testing sequence lengths that are required to make activation classifications across voxels depend on SNRs, which are functions of signal variability and activation strength. These ratios, even for a same paradigm, may differ from subject to subject, so savings are achieved by sequentially estimating parameters associated with activation on an individual basis. We will demonstrate these savings in simulations, and in a real-data example. First, we introduce basic theory of the SPRT in the context of fMRI analysis and then describe the adopted statistical framework for dynamically analyzing BOLD signal and associating task to activation status.

## Theory

### 1. Wald’s Sequential Probability Ratio Test

The general procedure of Wald’s SPRT is described as follows. Consider the hypothesis, *H*_*0*_: *θ = θ*_*0*_ against *H*_*a*_: *θ ≥ θ*_*1*_, where *θ*_*1*_ is a value greater than *θ*_*0*_ and is considered as practical different with *θ*_*0*_, *θ*_*1*_ *- θ*_*0*_ *≥* 0. Implementation of Wald’s SPRT starts by computing Wald’s likelihood ratio statistic after observing data [10]:

$$\Lambda_{t}=\log\left(\frac{f\left(Y_{t}|\theta_{1}\right)}{f\left(Y_{t}|\theta_{0}\right)}\right)$$

where ***Y***_***t***_ denotes the *t* observations up to a time *t*, *y*_*1*_ to *y*_*t*_. *f(* ***Y***_***t***_ *|θ*_*0*_ *)* and *f(* ***Y***_***t***_ *|θ*_*1*_ *)* are the respective one-parameter probability densities functions of ***Y***_***t***_ given *θ*_*0*_ or *θ*_*1*_ is the true value of parameter of interest. After *y*_*t*_ is observed at a time point t, one of three possible decisions is made according to the following rules:

1. Continue sampling if B < Λ_t_ < A
2. Stop sampling and accept H_0_ if Λ_t_ < B
3. Stop sampling and accept H_a_ if A < Λ_t_,

where stopping boundaries (A,B) = (log((1-β)/α), log(β/(1-α))), and Type I and Type II error are respectively denoted as α and β. These values are specified before testing.

In this setting, normally distributed errors will be assumed, so that the SPRT has the monotonic likelihood ratio property. Thus, if *θ > θ*_*1*_, the test will have even more power to detect the alternative, and shorter scan times will generally be required to detect the alternative. A modification of the original SPRT formulation for stopping is to consider the truncated SPRT [11], which will additionally call for stopping if an upper bound for the number of observations is reached. In practice, such a modification is needed, as theoretically it is possible for testing to continue indefinitely.

There are three attractive aspects to Wald’s SPRT. First of all, the boundary of Wald’s SPRT is only related to the pre-specified Type I and Type II error levels. Hence, the boundary can be computed without assumptions being made about the underlying distribution of the data, so that SPRT can be applied under general conditions. Secondly, Wald’s SPRT is designed to satisfy not only Type I error (= α) but also statistical power (= 1 - β). Most fixed sample design tests only control Type I error. We will see in a subsequent simulation that there can be much higher level of Type II errors in traditional analysis approaches compared with SPRT-based methods when identifying activation status of voxels. Thirdly, Wald’s SPRT is an efficient test because required sample sizes can be much less than fixed designs that have similar classification accuracy properties. These properties approximately hold for the truncated version as well, particularly if the upper bound is a large value.

We propose an approach based on combining information obtained through parallel voxel-level (truncated) SPRTs to inform real-time adjustments in fMRI experimental designs according to observed rtfMRI signal. Two common research objectives in fMRI are to 1) identify regions with activation associated with specific tasks; and 2) identify regions with differential activation in the sense higher activation levels are associated with one task over another. These objectives can be studied and analyzed through formulation of statistical hypotheses, such as by linear contrasts of regression coefficients. In simulations, the efficiency and accuracy of the proposed sequential methods for conducting a range of different hypothesis tests is demonstrated by comparing required fMRI scan volumes and voxel-level classification accuracy rates with the conventional method based on fixed designs. Moreover, the proposed methods can assess hypotheses related to activation with either one or two-sided hypothesis tests. Hypothesis tests in a range of simulated scenarios will be considered. First, details of the proposed approach are described in the following section.

### 2. Voxel-wise general linear model in fMRI

#### General Linear Model

The most common fMRI statistical analysis approach is by using voxel-wise GLM, to see how well observed BOLD response associates with expected BOLD response from stimuli [12]. Voxels that are activated by a task are identified through conducting statistical inference on a task-related regression parameter. For a given voxel, the GLM is:

$$Y_{t}=XB+E_{t}$$ (1)

where ***Y***_*t*_ is a t × 1 vector of measured BOLD signal intensities of the voxel over time, with y(n) denoting observed BOLD response at time n, 1 ≤ n ≤ t. ***X*** is a t × (P + 1) design matrix that represents the expected BOLD signal change per task for P tasks, generated by convolving a hemodynamic response function (HRF) with variables that indicate the timing of when a respective task is administered. A commonly used canonical HRF is the double gamma HRF, which we will consider below in all analyses. Specifically, for a task p, *x*_*p*_(n) represents the expected observed BOLD response at time n due to administration of task p. Additionally, a first column of 1’s is included for an intercept term. Thus, ***B*** equals [*b*_0_ *b*_1_···*b*_*p*_···*b*_*P*_]^*T*^, a (P + 1) × 1 regression coefficients vector that includes an intercept *b*_0_, as well as task-related parameters *b*_*p*_, p = 1 … P. In this formulation, *b*_*p*_ respectively represents the magnitude of HRF activation associated with task *p*. The *t×*1 error vector, ***E***_*t*_, represents the error (or noise) component, and is assumed to be normally distributed with mean zero and variance *σ* ^*2*^ ***V***_*t*_. Note that *σ* ^*2*^ is the error variance and ***V***_*t*_, a *t*×*t* matrix, represents the temporal autocorrelation structure. Thus, ***Y***_*t*_ is assumed to have a multivariate normal probability distribution, as follows:

$$f\left(Y_{t},B,\sigma^{2}V_{t}\right)=\frac{1}{{\left(2\pi \right)}^{T/2}\left|\sigma^{2}V_{t}\right|}\exp\left(-\frac{1}{2}\left(Y_{t}-XB\right)'{\left(\sigma^{2}V_{t}\right)}^{-1}\left(Y_{t}-XB\right)\right)$$

where |*σ* ^*2*^ ***V***_*t*_| is the determinant of *σ* ^*2*^ ***V***_*t*_. Major sources of noise in fMRI data that are represented by ***E***_*t*_ include brain metabolism and physiology, and spontaneous fluctuations. These sources can involve temporal autocorrelation, so that a temporal autocorrelation structure is considered. Using generalized least square (GLS) estimation of regression parameters, one is able to make inferences about a voxel’s activation status through testing of hypotheses.

The quality of signal data for detecting activation for a specific task can be indicated by a voxel-wise calculation of time series SNR, which is defined here as follows (see also [13]):

SNR = (Beta value associated with task) / (within voxel standard deviation of error, *σ*)

#### Conventional fixed designs versus sequential designs

In conventional analyses based on fixed designs, parameters are estimated only after acquisition of all images is completed. In contrast, in sequential testing, estimates of ***B*** are computed and updated after each scan volume is observed. Since the focus of experimentation is to obtain these estimates for inference, this updating process allows for real-time decisions to be made on how to adjust the experimentation dynamically. For instance, as we will describe next, based on the updated estimates and their statistical properties, decisions are made using SPRT for whether or not to continue administration of a task. The likelihood functions that comprise the SPRT rely on the same statistical models and assumptions as for the fixed design model, and hence the same statistical parameter estimation approaches. Inferentially, in the sequential designs to be proposed below, focus is given on hypotheses that assess whether the associated task parameter in the regression model is zero or greater than or equal to some predetermined value *δ >* 0. This differs from the standard alternative GLM hypotheses, in that the alternative assumes a minimum difference from zero. Ideally, this threshold represents a significant clinical difference. In the examples below, we select these values based on projected SNR associated with the alternative hypothesis threshold value (i.e. standard deviation units).

#### One-sided Voxel-wise SPRT

We first consider SPRT for one-sided hypothesis tests on contrasts of voxel-specific regression parameters from GLM models. Wald’s SPRT methods can be implemented with either independent and identically distributed (i.i.d.) data or on non-i.i.d. data [14]. Two-sided analogues are similar, as will be seen later on. The general form of one-sided hypotheses is

$$H_{0}:cB-\theta_{0}=0versusH_{a}:cB-\theta_{0}\geq \delta$$

where ***c*** equals [*c*_1_—*c*_*p*_—*c*_*P*_], a (P + 1) × 1 contrast vector. ***cB*** is a linear combination of corresponding coefficients. *δ* is considered with practical important difference from *θ*_*0*_. For instance, the hypothesis test of the form *H*_*0*_: *b*_*p*_ *= 0* against *H*_*a*_: *b*_*p*_ *≥ 1* represents the test of whether or not a voxel is activating in association with a task *p*, assuming that a magnitude of 1 is considered a practical difference from 0. Other comparisons of task activation magnitudes can be represented by linear combinations of regression parameters associated across several tasks, which we illustrate in the simulations.

$c\hat{B}$, the least squares (or maximum likelihood) estimate, is assumed to be normally distributed with mean ***cB*** and variance$Var\left(c\hat{B}\right)$. It takes the form:

$$c\hat{B}=c\left({\left(X'V_{t}X\right)}^{-1}X'V_{t}Y_{t}\right)$$ (2)

The statistic that is the basis for a one-sided SPRT is a likelihood ratio of $c\hat{B}$ *= θ*_*1*_ given observations collected up to a time point t divided by the likelihood of $c\hat{B}$ *= θ*_*0*_ given the same observations. The formula of likelihood ratio [14,15] is:

$$\begin{array}{l} \Lambda_{t}=\log\left(\frac{f\left(c\hat{B}|\theta_{1}\right)}{f\left(c\hat{B}|\theta_{0}\right)}\right)\\ =\log\left(\frac{\frac{1}{{\left(2\pi \right)}^{1/2}{\left|Var\left(c\hat{B}\right)\right|}^{1/2}}\exp\left(\frac{-1}{2}\left(\left(c\hat{B}-\theta_{1}\right)'Var{\left(c\hat{B}\right)}^{-1}\left(c\hat{B}-\theta_{1}\right)\right)\right)}{\frac{1}{{\left(2\pi \right)}^{1/2}{\left|Var\left(c\hat{B}\right)\right|}^{1/2}}\exp\left(\frac{-1}{2}\left(\left(c\hat{B}-\theta_{0}\right)'Var{\left(c\hat{B}\right)}^{-1}\left(c\hat{B}-\theta_{0}\right)\right)\right)}\right)\\ =\frac{1}{2}\left\{\left(\left(c\hat{B}-\theta_{0}\right)'Var{\left(c\hat{B}\right)}^{-1}\left(c\hat{B}-\theta_{0}\right)\right)-\left(\left(c\hat{B}-\theta_{1}\right)'Var{\left(c\hat{B}\right)}^{-1}\left(c\hat{B}-\theta_{1}\right)\right)\right\} \end{array}$$ (3)

However, Wald’s original formulation of SPRT doesn’t account for unknown “nuisance” parameters that are not the main focus of inference, in this case$Var\left(c\hat{B}\right)$. Estimates of such values will consequently be assumed as true values in the calculation of the SPRT, following as in Cox [16]. These unknown parameters can vary between subjects. For example, the variance, ***$Var\left(c\hat{B}\right)$***, may differ from person to person and even change across various fMRI experimental designs. Therefore, according to Cox’s work [16], $Var\left(c\hat{B}\right)$is replaced by corresponding maximum-likelihood estimations (MLEs) computed by the following equations:

$$\hat{Var\left(cB_{t}\right)\;}\;=\;{\hat{\sigma }}_{t}^{2}\times c{\left(X'V_{t}X\right)}^{-1}c'$$ (4)

where ${\hat{\sigma }}_{t}^{2}$ is the MLE for *σ* ^2^ up to t observations. For the scope of this investigation, the temporal autocorrelation structure, ***V***_***t***_, will be assumed known, for computational simplicity. Note that these variances are estimated at the voxel level.

Given this SPRT, stopping criteria at the single voxel level are as follows. Asymptotically, due to consistency in the estimators of unknown parameters used in Cox’s SPRT, the same horizontal stopping boundaries as for Wald’s SPRT can be employed [15]. Based on user specified values of Type I error α and Type II error β, the decision is made by following rejection/acceptance rules after collecting each scan image. Let *T* > 0 denote the upper bound. Supposing up to scan time *t < T* has been observed, decision rules for proceeding are defined as:

1. Continue sampling when B < Λ_*t*_ < A
2. Stop sampling and accept *H*_*0*_ when Λ_*t*_ < B
3. Stop sampling and accept *H*_*a*_ when A < Λ_t_

where stopping boundaries (A,B) = (log((1-β)/α), log(β/(1-α))). Note if *t = T*, then sampling is stopped, and *H*_*1*_ is accepted if Λ_*t*_ > 0; otherwise, accept *H*_*0*_. (5) Certainly, in practice, multiple voxels that comprise the ROIs will be analyzed simultaneously. The determination of whether to stop fMRI scanning for an experiment should involve jointly considering all these voxels. A global stopping rule will be proposed below that takes into account how many of the voxel-level analysis call for stopping, so these single voxel stopping rules are still pertinent to the overall goal of developing sequential methods for fMRI analyses.

#### Two-sided Voxel-wise SPRT

It may also be of interest to test two-sided hypotheses about values of ***cB***, such as of the form:

$$H_{0}:\left|cB-\theta_{0}\right|=0versusH_{a}:\left|cB-\theta_{0}\right|\geq \delta$$ (6)

where a difference between ***cB*** and *θ*_*0*_ greater than *δ* is considered as practically important.

As an example, suppose ***cB*** = b_1_—b_2_, where b_1_ and b_2_ respectively reflect activation levels for task 1 and 2. It may not be known a priori for which task activation may be higher for the voxel, and hence a two-sided hypothesis would be appropriate. Given normality of the error terms, the estimate $c\hat{B}$ is assumed normally distributed with mean of ***cB*** and$Var\left(c\hat{B}\right)$. Let δ> 0 be as in (6). The likelihood given the alternative hypothesis is true is a weighted average of *f(* ***Y***_*t*_ *|$c\hat{B}$* ***=*** *θ*_*0*_ *- δ)* and *f(* ***Y***_*t*_ *|$c\hat{B}$* ***=*** *θ*_*0*_ *+ δ)*. An equal weight of 1/2 has been shown to be the optimum weighting amount [10,17]. The SPRT statistic for two-sided hypotheses for voxel-level analysis is:

$$\begin{array}{l} \Lambda_{t}=\log\left(\frac{\frac{1}{2}\left(f\left(c\hat{B}|\theta_{0}-\delta \right)+f\left(c\hat{B}|\theta_{0}+\delta \right)\right)}{f\left(c\hat{B}|\theta_{0}\right)}\right)\\ =\log\left(\frac{\begin{array}{l} \frac{1}{2}\left(\frac{1}{{\left(2\pi \right)}^{1/2}{\left|Var\left(c\hat{B}\right)\right|}^{1/2}}\exp\left(\frac{-1}{2}\left(\left(c\hat{B}-(\theta_{0}+\delta )\right)'Var{\left(c\hat{B}\right)}^{-1}\left(c\hat{B}-(\theta_{0}+\delta )\right)\right)\right)\right)+\\ \left(\frac{1}{{\left(2\pi \right)}^{1/2}{\left|Var\left(c\hat{B}\right)\right|}^{1/2}}\exp\left(\frac{-1}{2}\left(\left(c\hat{B}-(\theta_{0}-\delta )\right)'Var{\left(c\hat{B}\right)}^{-1}\left(c\hat{B}-(\theta_{0}-\delta )\right)\right)\right)\right) \end{array}}{\frac{1}{{\left(2\pi \right)}^{1/2}{\left|Var\left(c\hat{B}\right)\right|}^{1/2}}\exp\left(\frac{-1}{2}\left(\left(c\hat{B}-\theta_{0}\right)'Var{\left(c\hat{B}\right)}^{-1}\left(c\hat{B}-\theta_{0}\right)\right)\right)}\right) \end{array}$$ (7)

Again, the SPRT statistic Λ_t_, including the statistical estimates that comprise it, are computed sequentially after each new fMRI scan image is collected (in other words, in real-time). Following Cox, $Var\left(c\hat{B}\right)$is replaced by the corresponding MLE computed as in (equation (4)). The decision is made by the same rejection/acceptance rules as for one-sided hypothesis tests, as in (5).

#### Multiple Comparisons Correction

One fMRI three-dimensional whole brain image volume can contain hundreds of thousands of voxels. Conducting voxel-wise analysis thus can include a very large number of hypotheses if each voxel is statistically considered separately, as we consider here. This presents what is known as the “multiple comparisons” issue, and care is needed in determining decision rules for hypotheses, in order to preserve simultaneous Type I and Type II errors. Bonferroni correction is used in this study, as these corrections are easily reflected in the stopping boundary specifications. The Type I and Type II errors for a single voxel’s hypothesis are modified as α_n_ = α/N and β_n_ = β/N, where N is the total number of hypotheses (voxels) being considered at once [18], which often may involve focus on a specific ROI.

#### Global Stopping Rule

Since we are possibly testing a large number of voxels at once, a “global” decision is needed on when to stop administration of a stimulus, based on aggregate performance of SPRTs across the voxel-wise tests. A well-known irony (or drawback) associated with SPRTs is that the largest number of observations required for stopping occurs precisely when we may be most indifferent to the actual parameter value being tested [17]. For instance, suppose that the true parameter value is half way between the null and alternative hypothesis values. This presents the most difficult problem in terms of number of scan volumes that will be required to stop in a classical SPRT. Yet, it also is the case where indifference to the classification results may be the greatest, as the value truly is “in-between” the null and alternative. For efficient stopping, we suggest a rule that stops when a pre-determined, user-defined percentage of voxel-level tests satisfy the stopping criteria specified as in (5). This allows for circumventing the waiting for voxel-level tests that are “stragglers” when the more clear-cut cases are already decisively decided upon as active or non-active. Therefore, a global stopping rule is proposed that allows for identification of the most distinctive voxel activation levels, but leaves some uncertainty for “borderline” cases that require relatively much more testing, and for which the consequences of misclassification are less. In return, as we will demonstrate, this allows for the chance to obtain substantial overall reductions in the number of scan volumes needed for activation determinations across an ROI. A key to success for this approach is to choose a pre-determined percentage that is reflective of the number of voxels with activation levels that don’t lie between the null and alternative hypotheses. The procedure for implementing a global stopping rule is:

1. Predetermine a targeted percentage level, denoted by *G*%, that is acceptable in terms of voxels that will have decisive classification
2. Stop fMRI scanning if at least *G*% of voxels satisfies (5) after multiple comparisons adjustment via Bonferroni correction. Otherwise, continue scanning.
3. At the stopping time point, T, the final decision on activation status for each of the voxels is made according to the following rules:
4. Accept *H*_*0*_ when Λ_T_ ≤ 0
5. Accept *H*_*a*_ when Λ_T_ > 0

#### Summary of voxel-wise SPRT procedures

In sum, the procedures of voxel-wise SPRT are recursively employed until a globally-determined stopping point for experimental administration is reached. The following steps are:

- **Step 1**: Collect one new fMRI image.
- **Step 2**: Across voxels, apply real time pre-processing procedures, such as spatial smoothing and normalized drift correction [19].
- **Step 3**: For each voxel-level GLM, compute MLEs of ***cB*** and $Var\left(c\hat{B}\right)$ based on equations (2) and (4).
- **Step 4**: For each voxel, compute an SPRT statistic Λ_t_, based on (equation 3) or (7), depending on the form of the hypothesis test, and the corresponding MLEs from Step 3.
- **Step 5**: Determine if stopping would be invoked for each voxel-level test based on rejection/acceptance rules as in (5), incorporating Bonferroni correction as needed.
- **Step 6**: Assess the global stopping criterion for the pre-determined target *G*%, to determine if stopping of the administration of the stimulus should be invoked. If not, repeat from Step 1.
- **Final Step**: If the specified global stopping is rule satisfied, at each voxel all the fMRI signal data that has been collected up to stopping for that voxel is used to make a final, determination as to activation status. The likelihood ratio as in (3) for one-sided hypothesis or (7) for two-sided hypothesis will be computed, and rule for deciding between hypotheses is to select the associated hypothesized parameter value with the largest corresponding likelihood value.

## Methods and Results

### 1. Simulation studies

In simulations we explore whether the proposed approach of conducting simultaneous voxel-wise SPRTs is able to achieve similar accuracy compared to a fixed, pre-determined experimental design, with the conventional fMRI analyses method through GLM, while at the same time significantly reducing scanning times. R package “nruRosim” [20] was used to generate simulated fMRI images. The simulated datasets were analyzed within the Matlab environment (64-bit version R2012a The Mathworks, Natick, MA).

In the simulations, the fixed experimental design includes two tasks, task A and task B. Each task block is presented for 4 seconds and then a rest block is presented for the next 20 seconds. The two tasks are presented by turns. One rest block is applied in the beginning of the experimental design. The block paradigm is thus given as the order of following sequence: R|A|R|B|R|A|R|B|R|…

where R represents a rest block and A and B respectively represent task A block and task B block. Each voxel has its own simulated fMRI signal. The simulated fMRI signals were generated by combining time series associated with activation activity and noise. Following the notation in (1), *P*, the total number of tasks performed in the simulated experimental design, is equal to 2 in this simulation. The terms in ***E***_***n***_ are generated by taking account of white noise, low frequency drift, physiological noise, temporal correlation and spatial correlation. White noise is assumed to be normally distributed. Low frequency drift is generated by a basis of discrete cosine functions. Heart beat and respiratory rate were separately set as 1.17 Hertz and 0.2 Hertz. Temporal correlated noise is generated based on an autoregressive order one (AR(1)) model with a *ρ* value of 0.3, which is suggested for TR = 2 seconds [21]. Spatial correlation is also modeled by an AR(1) process with *ρ* value of 0.7.

**Fig 1:**
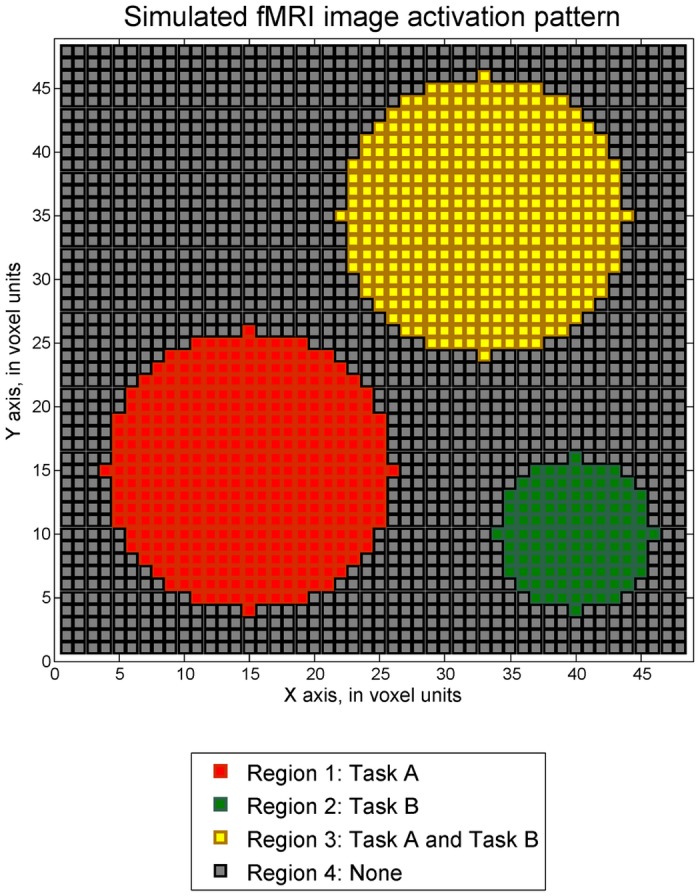
Activation pattern of simulated fMRI image.

Our simulations will be structured as follows. Two fMRI image scenarios within a ROI are simulated. Each image has three activation regions, as shown in Fig. 1. One is simulated with relatively lower maximum SNR of 0.1, and the other one is simulated with maximum SNR values of 0.3. Both simulated images have a size of 48 × 48 voxels. The activation pattern includes three circular shaped regions 1, 2 and 3 with exponential decay activation responses which are separately activated by task A only (*b*_*1*_ *= β > 0*, *b*_*2*_ *= 0*), task B only (b_*1*_ *= 0*, *b*_*2*_ *= β > 0*) or both task A and task B (*β*_*1*_ *= β >0*, *β*_*2*_ *= β*) shown in Fig. 1. Region 1 includes 377 voxels, region 2 includes 113 voxels and region 3 includes 377 voxels. They are separately 16.36%, 4.9% and 16.36% of the total number of voxels (2304) in one simulated image. Region 1 is designed to explore the detection on a bigger size ROI and region 2 is for smaller size ROI detection. Region 3 is used for investigating detection when activation occurs for both tasks, including differential activation. Simulated image with maximum SNR of 0.1 is generated by defining the maximum *b*-values of the activation regions as equal to 1 (*b*_1_ = *b*_2_ = 1) and another image with maximum SNR of 0.3 is generated by defining the maximum *b* values of the activation regions as equal to 3 (*b*_1_ = *b*_2_ = 3). The remaining *b*-values for voxels in activation regions are exponentially decaying to zero from the peak value. A sequence of simulated images is generated based on the respective set of parameter values at each voxel, for both task activation and noise.

Before the simulated images were analyzed by either voxel-wise SPRT or with a fixed sample design, two steps of pre-processing procedures, spatial smoothing and normalized drift correction were applied. Spatial smoothing is with a Gaussian kernel, 6 full width at half maximum (FWHM) weighting on 3 by 3 voxels [22], and a normalized drift correction method (see [19]; correction was done with parameter value 0.1). The pre-processing steps involving spatial smoothing and drift correction are feasible for real time.

Four simulation experiments were performed: 1) Simulated images with maximum SNR of 0.1 were analyzed by one-sided voxel-wise SPRT for task A active regions in the first simulation analysis. 2) In the second simulation analysis, the same simulated images and same task A only activation are detected by two-sided voxel-wise SPRT. 3) Maximum SNR of 0.3 simulated images were analyzed for task A activation by one-sided voxel-wise SPRT in the third simulation analysis. 4) In the last simulation, maximum SNR of 0.1 simulated images were analyzed by one-sided voxel-wise SPRT to detect regions of differential activation between task A and task B. Finally, a fixed length of 360 scan volumes was used as the benchmark sample size in the traditional GLM analyses to follow. This value was selected because task activation detection at 360 scan volumes was found to have attractive accuracy levels, particularly for the first simulation that was considered. For reference, we adopt this fixed length for other analyses as well. The simulated and real data are available from the authors upon request.

#### 1.1 Simulation model

For one voxel with t scan volumes represented in time series data, the linear model in the simulation analysis, including intercept term and two task-related regressors. For the SPRT analysis, Type I error and Type II error are separately set as 0.01 and 0.1. In this analysis, suppose false positives are considered less desirable than false negatives. The stopping boundaries in the SPRT method are corrected for the multiple comparisons among voxels by the Bonferroni approach. In the fixed sample GLM analysis, the Bonferroni approach is too conservative to detect the active regions, as Type I error thresholds values are very small. Therefore, multiple comparison issue is corrected by controlling the false discovery rate (FDR) [23,24].

#### 1.2 Simulation results

##### Efficiency and accuracy of one-sided voxel-wise SPRT on activation detection

**Fig 2:**
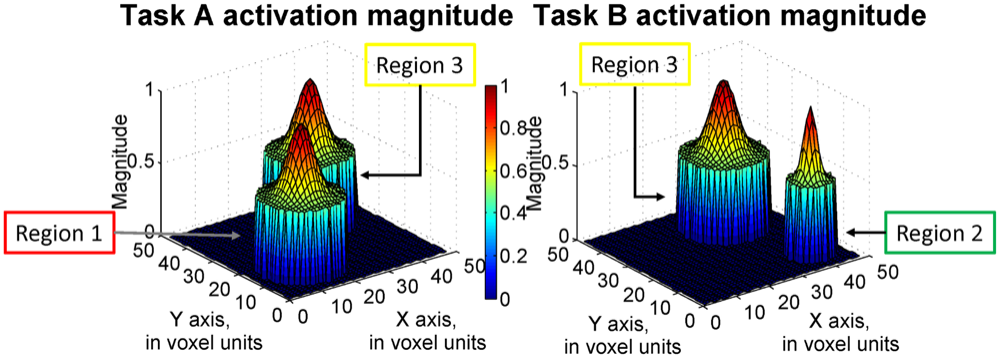
The activation magnitude structure of the dataset (maximum SNR = 0.1).

**Fig 3:**
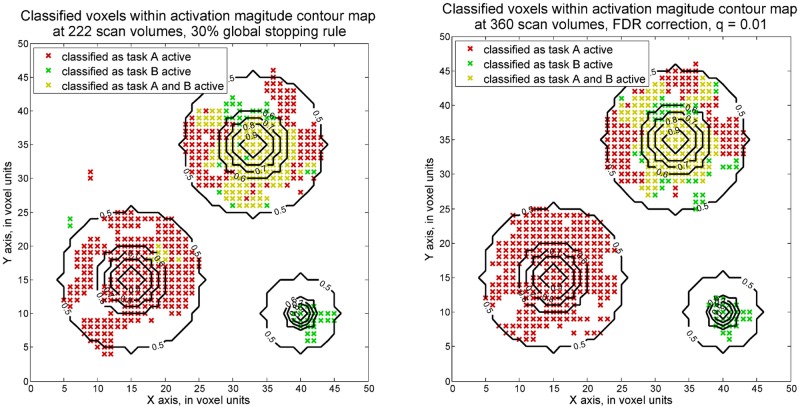
Voxels classified as active based on one-sided voxel-wise SPRT (maximum SNR = 0.1). The classification results of activation status by one-sided voxel-wise SPRT (left) and fixed sample GLM (right) were shown separately. Red x signs, green x and yellow x separately stand for voxels classified respectively as task A active, task B active and both tasks active. Activation strength contours are also labeled in these two plots, from 0.9 to 0.5. The thresholds for the voxel-wise SPRT are set through 0.01 Type I error and 0.1 Type II error and adjusted by Bonferroni correction approach. For the fixed sample GLMs, FDR correction approach was adopted, with q = 0.01.

The goal of the first simulation experiment we consider is to explore the efficiency of the proposed one-sided voxel-wise SPRT for high detection accuracy of the fMRI image when SNR is relatively weak. The hypothesis associated with task A activation is *H*_*0*_: ***cB*** *= 0* against *H*_*a*_: ***cB*** *≥ 1* where ***c*** equals [0 1 0] and the hypothesis of task B is *H*_*0*_: ***cB*** *= 0* against *H*_*a*_: ***cB*** *≥ 1* where ***c*** equals [0 0 1]. Here, activation strength level with value greater than or equal to 1 is considered as a practically significant value from zero in this study. A dataset with maximum SNR of 0.1 was generated and conventional fMRI analysis methods employed. The simulated activation strength structure of the analyzed dataset is displayed in Fig. 2. The maximum strength of activation is 1 and the rest of the voxels are active with decreasing magnitudes from the strongest voxel. Here we specified the ability on localizing the peak of activation regions. In our analysis, the voxels with greater than 0.8 activation strength are considered as located in the target areas, as these values are close to 1. Below, detection accuracy is presented as the percentage of voxels that is correctly detected among the voxels truly having greater than 0.8 magnitudes. For instance, the detection accuracy at region 3 is defined as the number of voxels correctly classified as both task A and task B active divided by the number of voxels with activation magnitudes greater than 0.8 for both task A task B. At region 4, the percentage of voxels correctly identified as inactive among truly inactive voxels is also calculated as the detection accuracy. The detection accuracy values from both sequential and fixed design method and for the four types of activation regions are presented in Table 1 and depicted in Fig. 3.

**Table 1:** Detection accuracies among the 4 simulated activation areas.

**A. Detection accuracies for the one-sided voxel-wise SPRT approach (30% global stopping, 222 scan volumes)**				
True activation strength	Region 1	Region 2	Region 3	Region 4
b = 0.0				99.37% (= 1428/1437)
1.0>b≥0.8	97.30% (= 36/37)	88.89% (= 8/9)	94.59% (= 35/37)	
**B. Detection accuracies for the one-sided, voxel-wise fixed sample GLM approach (360 scan volumes, FDR correction)**				
True activation strength	Region 1	Region 2	Region 3	Region 4
b = 0.0				99.93% (= 1436/1437)
1.0>b≥0.8	94.59% (= 35/37)	100% (= 9/9)	100% (= 37/37)	

Detection accuracies are within around 5% error for one-sided voxel-wise SPRT and voxel-wise, fixed sample GLM, except when 8 of 9 voxels within the higher activation range are correctly classified, leading to 88.89% accuracy.

Note that the one-sided voxel-wise SPRT is able to achieve around 95% detection accuracy among all four regions using a 30% global stopping rule and 222 scan volumes were required in total. Voxel-wise SPRT is thus able to achieve a similar accuracy as when 360 scan volumes are employed in the fixed design. This means that the proposed method needs around a 40% shorter scan time period to achieve comparably high accuracy than the conventional fixed design analysis.

##### Efficiency and accuracy of two-sided voxel-wise SPRT on activation detection.

**Fig 4:**
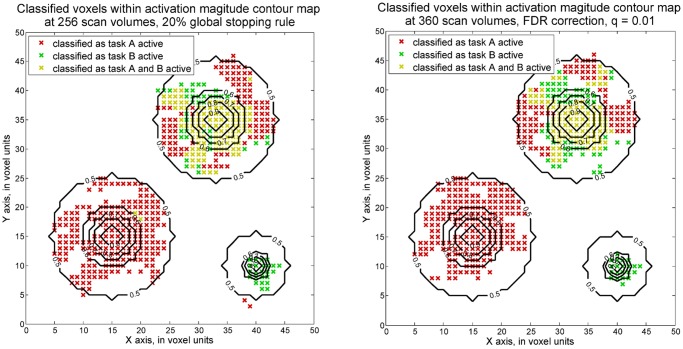
Voxels classified as active based on two-sided voxel-wise SPRT (maximum SNR = 0.1). The plot on the right shows the voxels classified as active by voxel-wise SPRT. The testing thresholds are 0.01 Type I error and 0.1 Type II error and adjusted by Bonferroni correction approach in terms of number of voxels in the ROI. Another plot shows the results from voxel-wise, fixed sample GLM, with q = 0.01 FDR correction applied.

It also may be of practical interest to consider two-sided hypotheses. Suppose *|b*_*k*_ *|>1*, k = 1 and 2, are considered as practically important differences. The simulated dataset with 0.1 maximum SNR for activated regions was again analyzed by sequential and fixed approaches. For two-sided hypotheses, in comparing the detection accuracies from voxel-wise fixed sample GLM, voxel-wise SPRT is seen to achieve comparable accuracy levels as with fixed designs by 256 scan volumes. Hence, around 30% saving is observed. The plots in Fig. 4 show how the voxels are identified as task A active, task B active or both task active by the two approaches. Corresponding detection accuracies of four regions are also presented in Table 2.

**Table 2:** Detection accuracies among four simulated activation areas, two-sided hypothesis.

**A. Detection accuracies using a two-sided voxel-wise SPRT approach (20% global stopping, 256 scan volumes)**				
True activation strength	Region 1	Region 2	Region 3	Region 4
b = 0.0				99.58% (= 1431/1437)
1.0>b≥0.8	94.59% (= 35/37)	100% (= 9/9)	91.89% (= 34/37)	
**B. Detection accuracies using a two-sided, voxel-wise fixed sample GLM approach (360 scan volumes, FDR correction)**				
True activation strength	Region 1	Region 2	Region 3	Region 4
b = 0.0				99.93% (= 1436/1437)
1.0>b≥0.8	94.59% (= 35/37)	100% (= 9/9)	94.59% (= 35/37)	

Two-sided voxel-wise SPRT and fixed sample GLM methods give comparable detection accuracies among four activation conditions. The proposed sequential methods required 57% less scan volumes.

##### Efficiency and accuracy of one-sided voxel-wise SPRT on activation detection with higher SNR.

**Fig 5:**
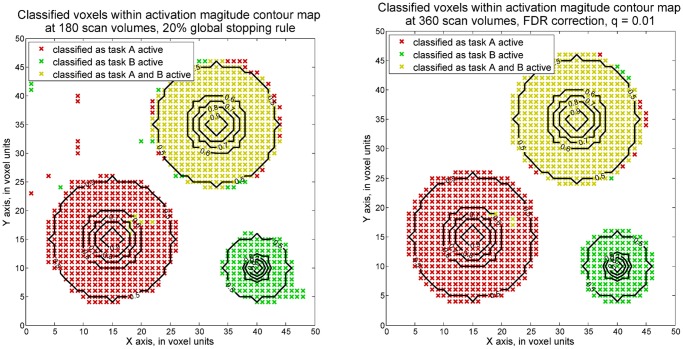
Voxels classified as active by one-sided hypothesis tests (maximum SNR = 0.3). The plot on the right shows the voxels classified as active by voxel-wise SPRT. Boundaries are derived for 0.01 Type I error and 0.10 Type II error, and corrected by the Bonferroni approach. The plot on the left shows the classified activation status generated by voxel-wise, fixed sample GLM, with q = 0.01 FDR correction applied.

In this section, a stronger task related signal dataset with 0.3 maximum SNR is assumed. This allows for investigation of the performance of SPRT when activation is actually stronger than the specified threshold in the alternative hypothesis. It is expected that the required sample size will be smaller. The hypothesis is still of the form *H*_*0*_: *b*_*1*_ *= 0* against *H*_*a*_: *b*_*1*_ *≥ 1* for detecting the task *A* activation and *H*_*0*_: *b*_*2*_ *= 0* against *H*_*a*_: *b*_*2*_ *≥ 1* for detecting the task B activation, since this is the assumed minimum difference of practical interest. However, the activation levels that were previously considered in the simulation with 0.1 maximum SNR are now assumed to have three times stronger task related signals. The results show that only 180 scan volumes are needed to identify with near 100% accuracy the voxels with activation magnitudes of 0.8 or greater in regions 1, 2 and 3. Region 4 has around 92% accuracy in detecting inactive voxels. Voxel-wise GLM also is able to achieve high accuracy across the four regions when the same fixed design of 360 scan volumes is adopted, as in the first simulation. Compared with traditional GLM, however, at least 50% scan volumes are saved by applying voxel-wise SPRTs. The results are presented in Fig. 5 and Table 3.

**Table 3:** Detection accuracies among four simulated activation areas.

**A. Detection accuracies for one-sided voxel-wise SPRT approach (20% global stopping, 180 scan volumes)**				
True activation strength	Region 1	Region 2	Region 3	Region 4
b = 0.0				92.69% (= 1332/1437)
1.0>b≥0.8	97.30% (= 36/37)	100% (= 9/9)	100% (= 37/37)	
**B. Detection accuracies for one-sided, voxel-wise fixed sample GLM approach (360 scan volumes, FDR correction)**				
True activation strength	Region 1	Region 2	Region 3	Region 4
b = 0.0				91.09% (= 1309/1437)
1.0>b≥0.8	100% (= 37/37)	100% (= 9/9)	100% (= 37/37)	

When the voxels have greater activation magnitude, activation status is easier to identify, and the required scan period is shortened through applying SPRT by 50%.

##### Efficiency and accuracy of one-sided voxel-wise SPRT on differential activation detection.

**Fig 6:**
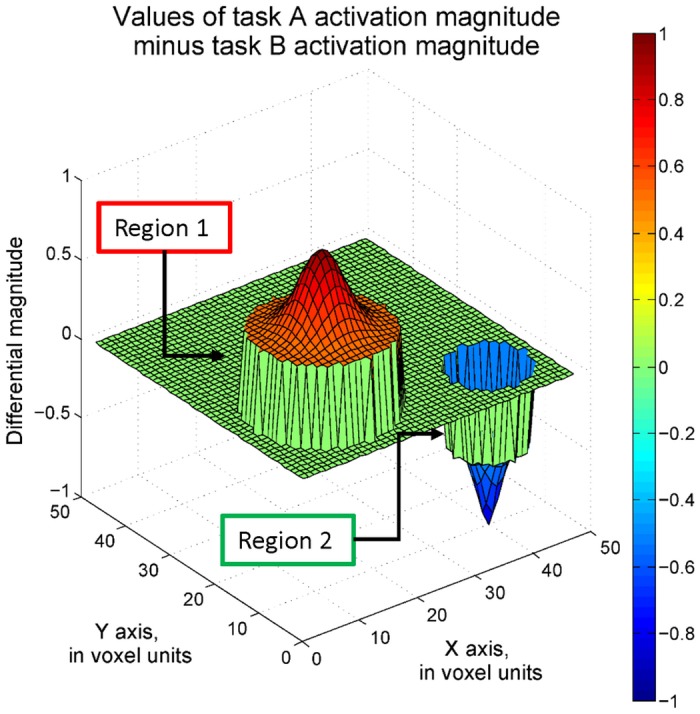
Differential activation magnitudes, with 0.1 maximum SNR.

**Fig 7:**
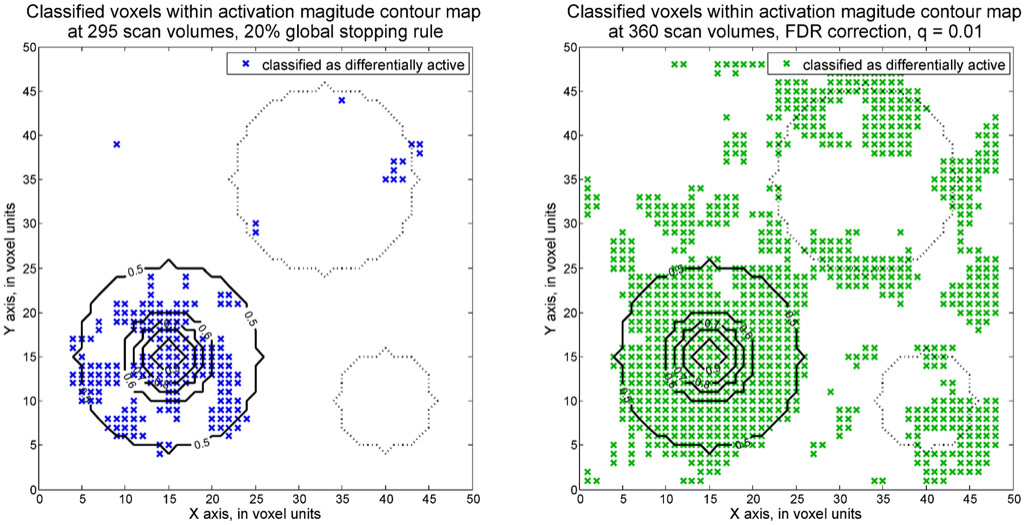
Voxels classified as differentially active by one-sided hypothesis tests (Maximum SNR = 0.1). The plot on the right shows the voxels with classified differential activation by voxel-wise SPRT. The thresholds are derived for 0.01 Type I error and 0.1 Type II error and corrected by the Bonferroni approach. The left side plot shows the result from voxel-wise, fixed sample GLM, with q = 0.01 FDR correction applied.

Voxel-wise SPRT is not only able to detect activated regions but also regions with differential activation. This can be achieved by testing a contrast of the task-related parameters. For instance, in order to detect the areas with higher task A activation magnitude than task B activation magnitude, a one-sided hypothesis can be set as *H*_*0*_: ***cB*** *= 0* against *H*_*a*_: ***cB*** *≥ δ*, some *δ >0*, where ***c*** equals [0 1–1]. Suppose a difference greater than 1 is considered practically important here, so that *H*_*a*_: ***cB*** *≥ 1*. The dataset with 0.1 maximum SNR was analyzed by voxel-wise SPRT and voxel-wise GLM here. The true differential activation structure is displayed in Fig. 6. Only region 1 has a positive difference of magnitude levels of at least 1 showing greater task A activity. Region 2, on the other hand, has a negative difference. There is no activation difference between task A and task B in regions 3 and 4. For the sequential and fixed design approaches, accuracies of identifying the voxels truly having greater than 0.8 differential activation magnitudes were computed, as in Table 4. In addition, the plots showing the voxels with classified differential activation are in Fig. 7. 295 scan volumes are needed by voxel-wise SPRT to achieve around 95% differential activation accuracy in region 1.

**Table 4:** Detection accuracies among four simulated differential activation areas.

**A. Detection accuracies for one-sided voxel-wise SPRT approach (20% global stopping, 295 scan volumes)**				
True activation strength	Region 1	Region 2	Region 3	Region 4
b = 0.0		100% = (113/113)	97.35% (= 367/377)	99.10% (= 1424/1437)
1.0>b≥0.8	94.60% (= 35/37)			
**B. Detection accuracies for one-sided, voxel-wise fixed sample GLM approach (360 scan volumes, FDR correction)**				
True activation strength	Region 1	Region 2	Region 3	Region 4
b = 0.0		61.95% (= 70/113)	64.19% (= 242/377)	71.82% (= 1032/1437)
1.0>b≥0.8	100% (= 37 /37)			

One-sided SPRT applied on a dataset with 0.1 maximum SNR. Note higher Type I error for fixed sample GLM.

Note for the fixed design, similar accuracy for region 1 is attained with 360 scan volumes. However, Type II errors are much higher in the other regions than compared with SPRT. In this case, the advantage of SPRT is distinct in terms of its ability to help insure attainment of overall statistical accuracy levels across regions.

### 2. Real fMRI studies

To demonstrate the potential impact of our proposed approaches with actual fMRI data, we consider the problem of identifying a well known and repeatedly described distinct region in the human ventral visual pathway, the fusiform face area (FFA), which is associated with the perception of faces, a distinct visual stimuli. The paradigm used has been previously reported in a recent publication [25]. Study protocol and procedures were approved by the Case Western Reserve University Institutional Review Board (IRB). For more details on study design, and data collection, please refer to [25].

#### 2.1 Material and Methods

##### Magnetic Resonance Imaging.

Structural and functional magnetic resonance imaging was conducted using a 4T Bruker-Siemens hybrid MR scanner. Structural images included an MPRage high resolution anatomical scan and T2-weighted anatomical scan. Functional images were obtained using an echoplanar imaging sequence with 38 contiguous slices, 3.8 by 3.8 in-plane resolution, TR = 2000 ms, TE = 20 ms, 90 degree flip angle). The echo time setting was selected to reduce drop out of signal near the sinuses due to magnetic inhomogeneity (which for instance affects the temporal lobes and orbitofrontal cortex). Under these settings, the experimental sequence has produced highly robust results, as reported in [25,26].

##### Stimuli and Task.

Participants underwent three fMRI runs, each lasting six minutes and 40 seconds. During each scan, the participants were instructed to look at a slideshow of pictures and rate how much they liked each picture on a 4-point scale. They were presented with one push button pad for each hand so they could make their response (strongly dislike, dislike, like, strongly like) by pressing the button with their left middle finger, left index finger, right index finger and right middle finger respectively. Each run consisted of 48 stimuli, each presented for six seconds followed by a jittered fixation slide of either zero, two, or four seconds. The experiment consisted of six conditions (adult faces, children's faces, houses, machines/computers, mature animals, youthful/cute animals). The analyses presented here only focus on adult faces and houses. The pictures were selected from online databases, cropped and resized in Adobe Photoshop to create a centered head-only version on a black background. All faces had a neutral expression.

The goal of the task was to ensure that participants were paying attention to the faces. It is know from decades of research on memory that asking for an affective judgment about a stimulus produces 'deep encoding', i.e. better memory due to thorough processing of the stimulus. Hence, the rating process was included to ensure participants attended to the stimulus for the majority of the relatively long stimulus presentation period.

##### fMRI data analysis.

In this event-related experimental design, adult face and house were displayed in random order with variable delay. The goal is to identify the adult face activation regions and differential activation regions that activate when shown an adult face stimulus but not when shown a house stimulus. We performed voxel-wise SPRTs of linear contrasts of GLM regression parameters. The experimental design matrix, ***X***, reflects one intercept term and has columns corresponding to each of the stimuli types, including those not specifically of interest here. Entries within these columns reflect expected observed HRF response convoluted with indicator variables indicating when a stimulus is administered. Each BOLD stack was then spatially smoothed with a Gaussian 3D filter with FWHM of 2 voxels (6mm).

The hypothesis of adult face detection is: *H*_*0*_: b_adult_face_ = 0 against *H*_*a*_: b_adult_face_ *≥* 1. This alternative hypothesis value was seen to correspond to an estimated maximum SNR of 0.1932, which is less than in our simulated scenario. The hypothesis of differential activation detection is: *H*_*0*_: b_adult_face_- b_house_ = 0 against *H*_*a*_: b_adult_face_- b_house_ *≥* 1. The contrast vector ***c*** such that ***cB*** = b_adult_face_- b_house_ respectively has the elements associated with adult face and house equal to 1 and-1, and all other entries equal to 0. Spatial smoothing was conducted as a pre-processing step. As an ROI, 717 voxels were selected as the focus the identification of FFA in the analysis by voxel-wise SPRT and GLM. This selection was based on FFA identification across previously analyzed subjects using the same paradigm.

#### 2.2 Real fMRI studies results

Real data is considered from a 20-year old female subject found to have successful (differential) activity localization from a prior GLM analysis involving the complete set of available scan data. Dynamic methods were applied to the existing data, and compared with the complete data findings of activation. The complete data was comprised of 200 scan volumes per session over the 3 sessions (600 in total, including 24 adult face stimuli and 24 house stimuli). Using voxel-wise SPRT, a dramatic saving in scan times was found with comparable localization findings.

**Fig 8:**
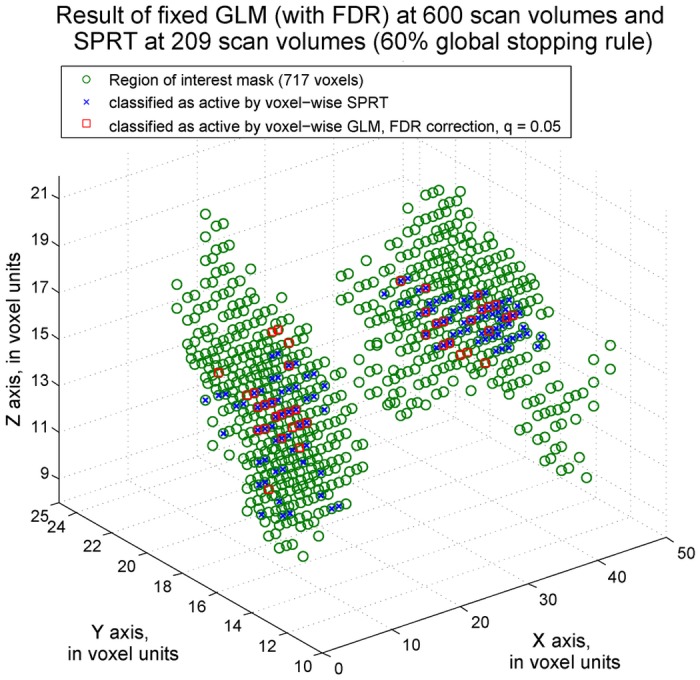
Adult face activity classification results. The voxels classified as activated by the adult face stimulus by voxel-wise fixed sample GLM methods are labeled by a red square. The voxels detected by voxel-wise SPRT are labeled as blue cross signs. q = 0.05 FDR correction was applied on fixed sample methods. SPRT bounds were derived for 0.01 Type I error and 0.1 Type II error, corrected by Bonferroni adjustment according to the number of voxels in the ROI.

We first considered a scenario where it is of interest to detect voxels that activate for the adult face stimulus. Within the ROI, only 160 scan volumes were required before a 60% global stop rule was satisfied. The complete data results determined that 328 voxels were active for the adult face stimuli, while the dynamic, SPRT-based approach found 399 such active voxels. There was an overlap of 303 voxels being found active with both approaches. See Fig. 8.

**Fig 9:**
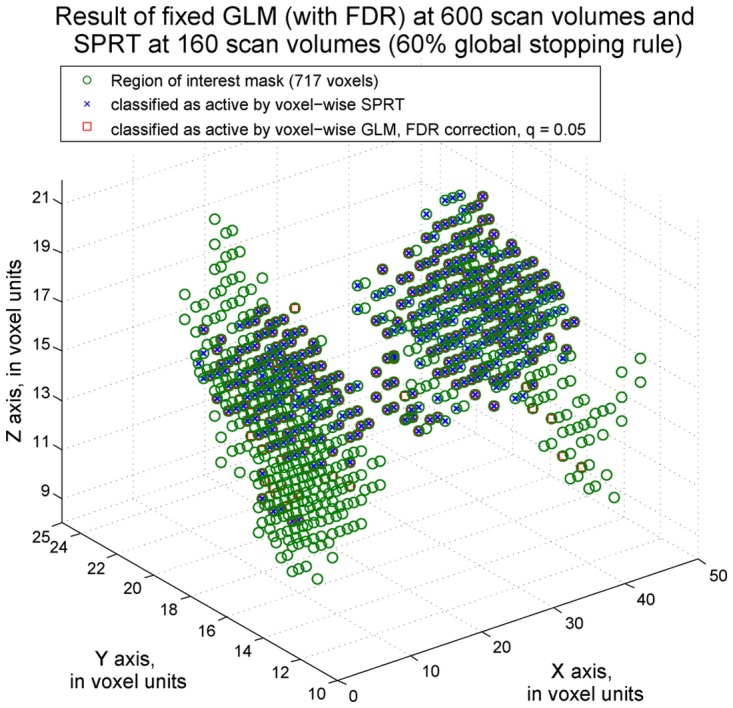
Differential activity detection classification results. The voxels classified as differentially active by voxel-wise fixed sample GLM methods are labeled as red square and the voxels detected by voxel-wise SPRT are labeled as blue cross signs. q = 0.05 FDR correction was applied on fixed sample methods. Boundaries derived for 0.01 Type I error and 0.1 Type II error, corrected by the Bonferroni approach, were adopted for the SPRT method.

For differential activation detection, 209 scan volumes were required through our proposed sequential methods. Overall stopping of administration of stimuli was again determined when more than 60% of the voxel-level SPRTs called for stopping individually. A traditional GLM approach applied on all 600 scans identified 88 voxels that are active for the adult face but not house image and voxel-wise SPRT detected 123 active voxels. 68 voxels were classified as active by both methods. The results are presented in Fig. 9. For activity and differential activity detection, the reductions in the sample size that are required by the SPRT method respectively are around 73% and 65% from 600 scan volumes. Hence, large savings were found. Moreover, there was a high level of overlapping results between approaches, indicating that the findings are still similar. Initially, Bonferroni correction was applied for the complete sample GLM analysis. However, this led to conservative results, in terms of low numbers of detections of active voxels. The results based on a FDR–based approach are displayed here to compare with results from voxel-wise SPRT. The notation q = 0.05 follows as in [24].

**Fig 10:**
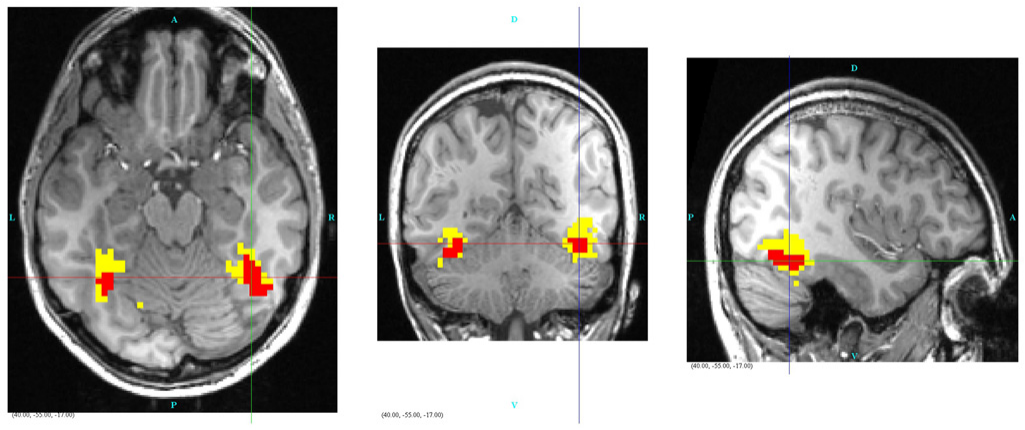
Illustration of differentially activated voxels (red), as determined by SPRT and the mask (yellow), overlaid on a T1-weighted image.

Finally, to facilitate visualization and localization of the mask and activated voxels within the brain, we display these areas in relation to the subject’s T1-weighted MRI. The example given in Fig. 10 is for the SPRT case and the identification of differentially activated voxels within the mask.

### 3. Dynamic adjustment of stimuli with SPRT

We now consider an application that illustrates how SPRT can be employed in the dynamic adjustment of stimuli. We consider the adjustment of task difficulty levels. Note that task-related activation in regions with a relatively low level of activation might be overlooked if a relatively strict threshold is applied in analyses [27]. In addition, various cognitive activation networks are used across individuals at different task-loading levels [28]. This means that across subjects, different activation patterns may arise even at similar difficulty levels. However, in most brain function investigations, only one task difficulty level is performed. This can be a source of incomparability among literature results, and leads to problems in decisively identifying neural processing regions. Moreover, in order to identify characteristics of cognition, it can be of interest to observe change in activation pattern over multiple task loadings. An example where this may be helpful is in characterizing notions of cognitive reserve.

Repeated clinical observations show that people with the same degree of brain pathology may have variant neuropsychological performances (NP) or clinical outcomes [29,30,31,32,33,34]. In 2002, Yaakov Stern proposed the concept of cognition reserve (CR) to explain this variability [35]. Stern postulated that there are differing capabilities for adapting brain networks of cognitive processes [36], and that more cognitive reserve leads to less clinical manifestation of impairment, even when given the same severity of brain damage. One aspect of CR is neural compensation [36,37]. Neural compensation refers to the phenomenon when alternative cognitive networks arise to cope with impaired brain networks.

#### Halving algorithm and SPRT for detecting neural compensation.

In detecting neural compensation for a given task, it may be that compensation is induced at different difficulty levels, depending on the individual. Such an instance is described in Stern 2012, where allowed time for response in a recall task was varied to adjust difficulty [28]. The goal of this example is to determine the minimum task difficulty level that will activate an ROI, such as one belonging to a neural compensation network. Again assume a block design for each level. Through dynamic adjustment of stimuli, it is not necessary to administer all difficulty levels, which can be time consuming. Instead, we propose a halving algorithm for dynamically selecting difficulty levels, that when used in conjunction with SPRT, can lead to substantial savings in scan volumes when identifying the minimum difficulty level for activation.

The approach of our proposed halving algorithm is to start from a mid-range difficulty level. Then, depending on whether or not activation is determined, based on BOLD responses observed in real time, a lower or higher difficulty level is administered. The assumption is that if a subject is not activating at a higher level, then that subject won’t be active at a lower one either. There would be no need, therefore, to test at relatively lower levels if activation is not observed. On the other hand, if activation is seen at the selected level, a lower level is administered next. Certainly, there is the possibility for error in the activation determinations, but we will see in a simulation study that classification performance can be quite good.

**Fig 11:**
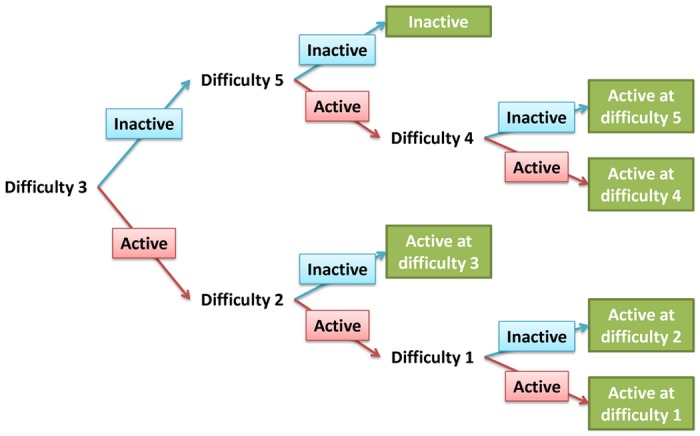
Halving algorithm for a task with five difficulty levels. Determinations of the minimum difficulty level when the target ROI starts to activate are denoted in green.

A halving algorithm is shown in a tree representation in Fig. 11 for a task with five difficulty levels, to represent adaptive selection sequences for difficulty level administration. As shown in Fig. 11, the implemented stimulus starts from a task with difficulty level 3, where the difficulty levels are ranked 1 through 5, from the easiest one to the hardest one. A harder or easier task is administered depending on the estimated task activation status for difficulty level 3. Applying the halving algorithm reduces the number of difficulty levels that must be administered from all five levels in a conventional fixed design to at most three. In conjunction, applying SPRT to determine activation status at each task difficulty level reduces the number of blocks that need to be administered even further.

#### 3.1 Simulation study

**Fig 12:**
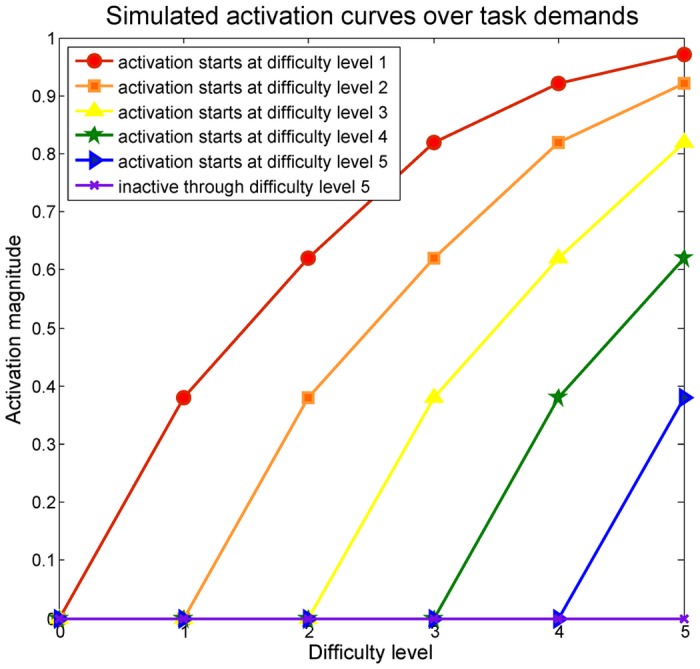
The simulated activation magnitudes, depending on the minimum difficulty level for when activation is initiated.

We generated activation response curves over the five task difficulty levels according to hypothesized activation curves from Stern’s investigations [36,38]. The corresponding activation magnitudes over task demand levels, as reflected by regression parameter values, are shown in Table 5. They serve as the basis for simulating subject responses, as shown in Fig. 12. The R package “nruRosim” [20] was used to generate simulated fMRI BOLD signals, and again, the simulated datasets were analyzed within the Matlab environment. For simplicity and illustrative purposes, in this example we consider time series consisting of one BOLD value per scan to represent an ROI, such as the mean of the voxel BOLD values in the ROI [39,40,41]. Although not studied here, these methods can be extended to SPRTs conducted on single voxel analyses across an ROI. For instance, an ROI can be considered activated if a certain percentage of voxels are declared activated, and global stop rules can be applied at each difficulty level.

**Table 5:** Hypothesized activation magnitudes for difficulty levels relative to when activation initially arises.

**Activation level**	**Activation amplitude (value of β)**
Inactive	0
Initial activation level	0.38
One level higher than initial	0.62
Two levels higher	0.82
Three levels higher	0.92
Four levels higher	0.97

In order to model realistic BOLD signals, a nonlinear Balloon model was used [42]. The simulated BOLD signals were produced by combining time series associated with activation activity and noise, as in (1). The corresponding activation amplitudes were defined as the regression coefficient values associated with the experimental design in GLM, as shown in Table 5. The thermal noise modeled by normal distribution was also added in the simulated BOLD signal. The SNR was kept to at most to 0.5 or below to fit clinical fMRI signal observation. One hundred individual signals were generated for each difficulty level being the minimum one.

In this simulation, at each difficulty level, the task was administered in a block design. Experimental blocks start with an 8-second rest period followed by a 3-second task stimulus. This cycle was repeated 30 times, ending with one 8-second rest period, for a total of 338 seconds or 169 scan volumes with a 2-second TR. For a design of this length, and given the simulated signal characteristics, accuracy in determining the true minimum activation length is seen to be fairly high.

#### 3.2 Simulation results

Again, the goal is to identify the difficulty level at which an individual starts to activate a specific ROI. Given a task with five difficulty levels, a traditional fixed design in this setting would require a total of 150 blocks. At each difficulty level, a GLM-based, one-sided hypothesis test of *H*_*0*_: b = 0 versus *H*_*a*_: b > 0 is conducted once all blocks have been administered, to make a decision on activation status. Consider the fixed design results, as displayed in Table 6. Each column represents the true minimum activity difficulty level, from 1 to 5, plus the inactive at any level case. Each row represents the classification decision made after observing the BOLD signals from 150 alternating cycles administered, using a GLM framework (t-test) at significance level α = 0.05. The last row shows the percentage of minimum difficulty levels of activation that were correctly classified from the 100 simulations per level. The accuracies are around 70% to 80%.

**Table 6:** Results from traditional GLM analysis.

**True level**						
**Classified level**	**1**	**2**	**3**	**4**	**5**	**Inactive**
1	**83**	7	6	3	6	2
2	17	**72**	6	4	4	4
3	0	21	**81**	5	3	4
4	0	0	7	**74**	2	7
5	0	0	0	14	**72**	1
Inactive at all levels	0	0	0	0	13	**82**
Accuracy (%)	83	72	81	74	72	82

The boldface numbers along the diagonal show the number of individuals whose minimum difficulty level for activation was correctly determined. In total, 150 blocks were administered to each subject for a given difficulty level.

For the sequential design involving the halving algorithm and SPRT, the decision rule can be denoted by the terminal node of the “path” taken in the tree representation shown in Fig. 11. At each non-terminal node, the path direction of the next stage is determined by the result of hypothesis test of *H*_*0*_: b = 0 versus *H*_*a*_: b *≥* δ = 0.38. This alternative hypothesis value is considered, due to small to the moderate SNR value associated with this value (σ^2^ = 1). The same signals as in the fixed design analysis were analyzed by SPRT. The results are displayed in Table 7. Columns represent the true difficulty level associated with initial activation, from 1 to 5, plus the inactive case. The average total number of blocks administered per individual is given in adjacent columns. Each row represents the classification decision made after dynamically observing the BOLD signal using the halving algorithm and SPRT, which are set at significance level of 0.05 and with Type II error of 0.10. The last row shows the percentage of correctly classified minimum difficulty levels among 100 simulations per actual difficulty level. The accuracies are around 70% to 90%. Note that our simulations show that sequential design can result in a savings of 72–83% in scan time over fixed designs, depending on a subject’s minimum level, while maintaining comparable accuracy.

**Table 7:** Results from proposed halving algorithms and voxel-wise SPRT.

**True level**	**1**		**2**		**3**		**4**		**5**		**Inactive**	
**Classified initially active level**		**Mean (Std) of required no. of alternative cycle**		**Mean (Std) of required no. of alternative cycle**		**Mean (Std) of required no. of alternative cycle**		**Mean (Std) of required no. of alternative cycle**		**Mean (Std) of required no. of alternative cycle**		**Mean (Std) of required no. of alternative cycle**
**1**	**83**	**30.27 (9.34)**	**2**	**56**	**0**		**0**		**0**		**0**	
				**(5.83)**								
**2**	**17**	**33.61 (11.70)**	**92**	**37.99 (11.13)**	**4**	**39.63 (21.69)**	**0**		**0**		**1**	**47.38**
												**(-)**
**3**	**0**		**6**	**27.63 (12.29)**	**83**	**29.05 (11.50)**	**8**	**29.12 (13.03)**	**5**	**25.28 (7.00)**	**4**	**36.09 (12.59)**
**4**	**0**		**0**		**12**	**34.27 (9.20)**	**82**	**37.28 (15.41)**	**5**	**35.15 (15.06)**	**0**	
**5**	**0**		**0**		**1**	**30.88(0)**	**10**	**41.98 (15.82)**	**72**	**41.61 (13.76)**	**3**	**47.83 (4.53)**
**Inactive**	**0**		**0**		**0**		**0**		**18**	**32.79 (13.61)**	**92**	**25.18 (11.55)**
**Accuracy**	**83%**		**92%**		**83%**		**82%**		**72%**		**92%**	

The bolded numbers on the diagonal line show the number of individuals whose initial activity level was correctly determined. The accuracy level among six initial activation groups was around 70% to 90%. The adjacent columns are the respective means of the number of blocks required for classification. *Std* represents standard deviation.

## Discussion

An important characteristic of voxel-wise SPRT is its ability to adaptively determine under pre-specified testing error thresholds when to stop administration of experimentation based on the observed data. Classification of activation status is made once sufficient evidence is collected in an aggregate manner across ROIs.

These results demonstrate that across a range of scenarios, voxel-wise SPRT can dynamically detect (differential) brain activation in an efficient and accurate manner. Even while often requiring substantially less scan volumes than fixed designs, comparably high accuracy levels are observed. It is expected that different combinations of task specific activation strength and error variability in the BOLD signal will be observed among different subjects, such as reflected by SNR measures. This affects required number of blocks needed for administration, so the flexibility provided by dynamic administration and stopping of stimuli is practically useful.

In contrast, for fixed experimental designs, in order to ensure precision for detection, there is a need for the experimentation length to be conservative, in that less favorable variance and activation level values must be assumed when determining amount of replication. This is necessary to help insure that scanning sessions will provide sufficient data for decisive statistical evidence for a wide range of subjects. Certainly, a major advantage of the proposed approach is that it can provide a great savings in cost, as it flexibly decreases the average time duration needed for a session. Importantly, accurate detections are consistently made from stopping only when predetermined Type I and Type II error levels are assured. Another advantage of being able to adjust and shorten an fMRI design is to minimize learning effects or fatigue.

The first simulation demonstrates the promise of this approach, in terms of savings in scan volumes while maintaining high accuracy in the simulated activation response surface. One-sided hypotheses are adopted across voxels, assuming that either there is increased magnitude of BOLD activation versus no activation. The proposed approach is not only appropriate for choosing between one-sided hypotheses but also two-sided hypotheses. Still, there are ramifications to sequential test length when specifying two-sided hypotheses. The numerator in SPRT statistics, the likelihood given the alternative hypothesis is true, is represented by weighted averages of two possible likelihoods. Because the numerator of the statistic is diluted by considering two ways of differentiation from the null hypothesis likelihood, less decisive evidence is provided by voxel-wise SPRT statistics. Therefore, the larger number of scan volumes is to be expected.

In the third simulation, the maximum SNR value is assumed to be higher than the level specified in the alternative hypothesis. This example gives illustration of performance for different SNR values than the one assumed in the first simulation, and how required test lengths may subsequently vary. Note that in practice, SNR values will vary across individuals, so that test length requirements will also vary. Here, we see that the required scan period for SPRT is shortened to 180 scan volumes when stronger signals (0.3 maximum SNR) are assumed. In the fourth simulation, the proposed method is demonstrated for more complex hypotheses, as represented by contrasts of regression parameters. In the example, the contrast being considered is the difference between two parameters, which represents whether differential activation is present. The use of sequential differential activation detection requires more scan volumes relative to the other hypothesis testing scenarios. Nevertheless, one-sided voxel-wise SPRT performs better in identifying non-differential activation than the fixed design, with high accuracy in regions 2, 3 and 4 (as shown in Fig. 7). In contrast, the accuracy levels for these regions suffer from higher levels of Type II errors as the fixed design length increases, since the focus of inference is on controlling Type I error in the standard GLM analysis. The proposed sequential approach, on the other hand, provides overall high accuracy of detection of both differential and non-differential activation.

A real data example confirms promise of this approach for activation and differential activation detection. In the real-data example, an objective is to identify regions that activate when shown an adult face stimulus but not when shown a house stimulus. This involved analysis of a contrast of regression parameters. Again, large saving in scan times of over 65% were observed using the sequential approach as compared with the length of the original design, even as comparable activation results were obtained. The overlap of voxels identified as active demonstrates that the sequential approach arrives at similar conclusions as the fixed design, but with much less scanning. Finally, we illustrate how SPRT can be used in conjunction with a halving algorithm to dynamically adjust stimuli, in the context of varying difficulty levels of a task. Even greater savings can be gained with use of SPRT in this example, as multiple decisions are being made with SPRT.

### Current limitations and areas for future research

From a sequential testing point of view, Bonferroni correction can be a very conservative approach to controlling the simultaneous Type I and Type II errors, especially in fMRI analysis where there are large number of voxel-level tests being conducted at once. Such adjustment can lead to very large or small significant threshold values and consequently, a large gap between the two stopping boundaries for each voxel-level test. This leads to a need for a large number of scan volumes, even with sequential testing. These methods may be more successful when focused ROIs are being studied, although the breadth of the simulations indicate that it may be possible to consider several moderately sized ROIs simultaneously, and still achieve efficiency gains while preserving statistical accuracy. Certainly, more than one hypothesis can be considered at once, with stopping criteria considered conjunctively, for instance. It should be noted that this would exacerbate the issue of multiple comparisons.

Further, temporal autocorrelation noise is generated by AR(1) model with a 0.3 ρ value in the simulated fMRI image. The signal intensity can be modified by pre-processing steps, such as spatial smoothing and normalized drift correction. Taking the 0.1 SNR simulated data as an example, the given 0.3 ρ value is reduced to 0.06 after two steps of pre-processing procedures. Therefore, ignoring temporal autocorrelation, one is still able to get high accuracy levels in the analysis. Further investigation displayed that the ρ values of noise terms including up to 0.7 in temporal autocorrelation decreased to maximum 0.12 after two steps preprocessing steps. However, the intensity of temporal correlation differs among various TR’s machines, experimental designs, and subjects [43]. Voxel-wise SPRT is able to include the temporal autocorrelation structure which is a possible source of noise in fMRI data [44]. A promising area of further research could involve establishing feasible real-time approaches to autocorrelation estimation that can demonstrate clear advantages in inferential accuracy [45].

The global stopping rule relies on expert opinion for selecting the appropriate percentage of voxels that have satisfied SPRT stopping criteria before stopping fMRI experiment administration. This percentage should exclude voxels for which a researcher would lie in-between the values in the hypotheses posed for the SPRT. As a future direction, a dynamic percentage selection rule that depends on real-time data could be developed to optimize testing objectives when determining when to stop.

The selection of the alternative beta value in the SPRT has ramifications when identifying activated voxels, and particularly when conducting multiple subject studies, and when there is a need to compare “apples with apples”. We have selected beta values in our hypotheses that lead to realistic signal to noise ratios, which assumes that the noise variance magnitude is approximately known. It may be possible to calibrate during experimentation the alternative beta value at the individual level, depending on estimated variance values, so that beta-values can be interpreted in terms of standard deviation volumes and SNR. This issue will be explored in future work.

Real time preprocessing procedures of fMRI such as drift correction, motion correction, temporal filtering, and spatial smoothing have been previously investigated [19,46]. As is well known, these steps may affect accuracy in signal detection. For instance, the implementation of spatial smoothing can decrease (or increase) activation magnitudes of voxels, especially those located at the boundary of active regions. This reduces the activation detection accuracy for the voxels located at the margins of active regions. However, these issues arise in conventional analyses as well.

Computational feasibility also is a concern, given the need for a large number of voxels to be analyzed simultaneously. Note that the matrix inversion in (2) (and as in (4)) is the most computationally burdensome step in the estimation and inference process. As was done here, it can be assumed that the estimation of regression parameters across voxels can share this same computation, as ***X*** can be the same across voxels. Hence, depending on assumptions and extent of ROIs, computational burden need not be a hurdle to actual real-time implementation. Initial tests in our development of an actual system using parallel processing are very promising, for the both parameter estimation and simple pre-processing steps.

Finally, the proposed approach can be extended to multi subject analyses by mapping results to Montreal Neurological Institute (MNI) or Talairach coordinate systems. Note that the statistical output after using SPRT still results in estimates of regression parameters and standard deviations at the voxel level. This basic statistical information can thus be translated per subject into a universal coordinates setting, even if experimental lengths vary across subjects, to facilitate comparison.

## Conclusion

In conclusion, voxel-wise SPRT shows promise for dramatically reducing scan time volumes in detecting activation compared to conventional fixed sample analysis. This is achieved through individualized sequential experimental designs, which adapt to a subject’s response to tasks, and allow for efficient stopping of fMRI stimulus administration. Immediate practical implications include reduced costs for fMRI sessions. Importantly, use of SPRT helps insure acceptable levels of statistical accuracy in activation determinations in terms of both Type I and Type II error. This work also serves as a fundamental basis for development of more complex and dynamic fMRI experimentation, and opens up new methodological challenges in fMRI data analysis.

## References

[1] Holman BL, Johnson KA, Gerada B, Carvalho PA, Satlin A (1992) The scintigraphic appearance of Alzheimer's disease: a prospective study using technetium-99m-HMPAO SPECT. J Nucl Med 33: 181–185. 1732438

[2] Bartsch AJ, Homola G, Biller A, Solymosi L, Bendszus M (2006) Diagnostic functional MRI: illustrated clinical applications and decision-making. J Magn Reson Imaging 23: 921–932. 16649199 10.1002/jmri.20579

[3] Ashby FG (2011) Statistical Analysis of fMRI Data: MIT press.

[4] Richard SJ, Frackowiak KJF, Frith CD, Dolan RJ, Price CJ, et al (2004) Human Brain Function: Academic Press.

[5] Cox RW, Jesmanowicz A, Hyde JS (1995) Real-time functional magnetic resonance imaging. Magn Reson Med 33: 230–236. 7707914 10.1002/mrm.1910330213

[6] Cohen MS (2001) Real-time functional magnetic resonance imaging. Methods 25: 201–220. 11812206 10.1006/meth.2001.1235

[7] deCharms RC (2007) Reading and controlling human brain activation using real-time functional magnetic resonance imaging. Trends Cogn Sci 11: 473–481. 17988931 10.1016/j.tics.2007.08.014

[8] Weiskopf N, Sitaram R, Josephs O, Veit R, Scharnowski F, et al (2007) Real-time functional magnetic resonance imaging: methods and applications. Magn Reson Imaging 25: 989–1003. 17451904 10.1016/j.mri.2007.02.007

[9] Wald A, Wolfowitz J (1948) Optimum character of the sequential probability ratio test. The Annals of Mathematical Statistics 19: 326–339.

[10] Wald A (1947) Sequential Analysis. New York: Wiley.

[11] Sawasd Tantaratana JBT (1977) Truncated sequential probability ratio test. Information Sciences 13: 283–300.

[12] Lazar N, editor (2008) The Statistical Analysis of Functional MRI Data Springer.

[13] Meyer FG, Shen X (2008) Classification of fMRI time series in a low-dimensional subspace with a spatial prior. IEEE Trans Med Imaging 27: 87–98. 10.1109/TMI.2007.903251 18270065

[14] Li JX (2010) Sequential probability ratio tests for generalized linear mixed models.

[15] Shah PK, Jeske DR, Luck RF (2009) Sequential hypothesis testing techniques for pest count models with nuisance parameters. J Econ Entomol 102: 1970–1976. 19886464 10.1603/029.102.0530

[16] Cox DR (1963) Large Sample Sequential Tests for Composite Hypotheses. Sankhyā: The Indian Journal of Statistics, Series A: 5–12.

[17] Govindarajulu Z (2004) Sequential Statistics: World Scientific Pub Co Inc

[18] De SK, Baron M (2012) Step-up and step-down methods for testing multiple hypotheses in sequential experiments. Journal of Statistical Planning and Inference 142: 2059–2070.

[19] Magland JF, Tjoa CW, Childress AR (2011) Spatio-temporal activity in real time (STAR): optimization of regional fMRI feedback. NeuroImage 55: 1044–1053. 10.1016/j.neuroimage.2010.12.085 21232612 PMC3057229

[20] Welvaert M, Durnez J, Moerkerke B, Verdoolaege G, Rosseel Y (2011) neuRosim: An R Package for Generating fMRI Data. Journal of Statistical Software 44: 1–18.

[21] Maus B, van Breukelen GJ, Goebel R, Berger MP (2011) Optimal design of multi-subject blocked fMRI experiments. NeuroImage 56: 1338–1352. 10.1016/j.neuroimage.2011.03.019 21406234

[22] Posse S, Fitzgerald D, Gao K, Habel U, Rosenberg D, et al (2003) Real-time fMRI of temporolimbic regions detects amygdala activation during single-trial self-induced sadness. NeuroImage 18: 760–768. 12667853 10.1016/s1053-8119(03)00004-1

[23] Genovese CR, Lazar NA, Nichols T (2002) Thresholding of statistical maps in functional neuroimaging using the false discovery rate. NeuroImage 15: 870–878. 11906227 10.1006/nimg.2001.1037

[24] Benjamini Y, Hochberg Y (1995) Controlling the False Discovery Rate—a Practical and Powerful Approach to Multiple Testing. Journal of the Royal Statistical Society Series B-Methodological 57: 289–300.

[25] Jack AI, Dawson AJ, Norr ME (2013) Seeing human: Distinct and overlapping neural signatures associated with two forms of dehumanization. NeuroImage 79: 313–328. 10.1016/j.neuroimage.2013.04.109 23657147

[26] Jack AI, Dawson AJ, Begany KL, Leckie RL, Barry KP, et al (2012) fMRI reveals reciprocal inhibition between social and physical cognitive domains. NeuroImage 66C: 385–401. 10.1016/j.neuroimage.2012.10.061 PMC3602121 23110882

[27] Zarahn E, Rakitin B, Abela D, Flynn J, Stern Y (2007) Age-related changes in brain activation during a delayed item recognition task. Neurobiol Aging 28: 784–798. 16621168 10.1016/j.neurobiolaging.2006.03.002

[28] Stern Y, Rakitin BC, Habeck C, Gazes Y, Steffener J, et al (2012) Task difficulty modulates young-old differences in network expression. Brain Res 1435: 130–145. 10.1016/j.brainres.2011.11.061 22197699 PMC3406734

[29] (2001) Pathological correlates of late-onset dementia in a multicentre, community-based population in England and Wales. Neuropathology Group of the Medical Research Council Cognitive Function and Ageing Study (MRC CFAS). Lancet 357: 169–175. 11213093 10.1016/s0140-6736(00)03589-3

[30] Katzman R, Terry R, DeTeresa R, Brown T, Davies P, et al (1988) Clinical, pathological, and neurochemical changes in dementia: a subgroup with preserved mental status and numerous neocortical plaques. Ann Neurol 23: 138–144. 2897823 10.1002/ana.410230206

[31] Price JL, Morris JC (1999) Tangles and plaques in nondemented aging and "preclinical" Alzheimer's disease. Ann Neurol 45: 358–368. 10072051 10.1002/1531-8249(199903)45:3<358::aid-ana12>3.0.co;2-x

[32] Crystal H, Dickson D, Fuld P, Masur D, Scott R, et al (1988) Clinico-pathologic studies in dementia: nondemented subjects with pathologically confirmed Alzheimer's disease. Neurology 38: 1682–1687. 3185902 10.1212/wnl.38.11.1682

[33] Morris JC, Storandt M, McKeel DW Jr, Rubin EH, Price JL, et al (1996) Cerebral amyloid deposition and diffuse plaques in "normal" aging: Evidence for presymptomatic and very mild Alzheimer's disease. Neurology 46: 707–719. 8618671 10.1212/wnl.46.3.707

[34] Mortimer JA, Snowdon DA, Markesbery WR (2003) Head circumference, education and risk of dementia: findings from the Nun Study. J Clin Exp Neuropsychol 25: 671–679. 12815504 10.1076/jcen.25.5.671.14584

[35] Stern Y (2002) What is cognitive reserve? Theory and research application of the reserve concept. J Int Neuropsychol Soc 8: 448–460. 11939702

[36] Stern Y (2009) Cognitive reserve. Neuropsychologia 47: 2015–2028. 10.1016/j.neuropsychologia.2009.03.004 19467352 PMC2739591

[37] Stern Y, Habeck C, Moeller J, Scarmeas N, Anderson KE, et al (2005) Brain networks associated with cognitive reserve in healthy young and old adults. Cereb Cortex 15: 394–402. 15749983 10.1093/cercor/bhh142 PMC3025536

[38] Stern Y (2007) Cognitive reserve: theory and applications. New York: Taylor and Francis xxi, 344 p. p.

[39] Friston KJ, Rotshtein P, Geng JJ, Sterzer P, Henson RN (2006) A critique of functional localisers. NeuroImage 30: 1077–1087. 16635579 10.1016/j.neuroimage.2005.08.012

[40] Saxe R, Brett M, Kanwisher N (2006) Divide and conquer: a defense of functional localizers. NeuroImage 30: 1088–1096; discussion 1097–1089. 16635578 10.1016/j.neuroimage.2005.12.062

[41] Etzel JA, Gazzola V, Keysers C (2009) An introduction to anatomical ROI-based fMRI classification analysis. Brain Res 1282: 114–125. 10.1016/j.brainres.2009.05.090 19505449

[42] Buxton RB, Wong EC, Frank LR (1998) Dynamics of blood flow and oxygenation changes during brain activation: the balloon model. Magn Reson Med 39: 855–864. 9621908 10.1002/mrm.1910390602

[43] Purdon PL, Weisskoff RM (1998) Effect of temporal autocorrelation due to physiological noise and stimulus paradigm on voxel-level false-positive rates in fMRI. Hum Brain Mapp 6: 239–249. 9704263 10.1002/(SICI)1097-0193(1998)6:4<239::AID-HBM4>3.0.CO;2-4 PMC6873371

[44] Yan C, Liu D, He Y, Zou Q, Zhu C, et al (2009) Spontaneous brain activity in the default mode network is sensitive to different resting-state conditions with limited cognitive load. PLoS One 4: e5743 10.1371/journal.pone.0005743 19492040 PMC2683943

[45] Feng IJ (2013) Dynamic adjustment of stimuli in real-time fMRI, doctoral dissertation, Case Western Reserve University.

[46] Caria A, Sitaram R, Birbaumer N (2011) Real-Time fMRI: A Tool for Local Brain Regulation. Neuroscientist. 10.1177/1073858411407205 21652587

