# Correction: Dynamic Adjustment of Stimuli in Real Time Functional Magnetic Resonance Imaging

**DOI:** 10.1371/journal.pone.0126192

**Published:** 2015-05-01

**Authors:** The PLOS ONE Staff

The following information is missing from the Funding section: This research was supported in part by Judy Polshek Goodman Alzheimer's Disease Research and Prevention Fund, and a pilot grant from Philips Imaging.

The complete, correct funding information is as follows: This research was supported in part by the Spitz Brain Health Innovation Fund, the G.R. Lincoln Family Foundation, Judy Polshek Goodman Alzheimer's Disease Research and Prevention Fund, and a pilot grant from Philips Imaging. The funders had no role in study design, data collection and analysis, decision to publish, or preparation of the manuscript.

The publisher apologizes for the error.
